# Factors associated with children and young people’s mental health in the English-speaking Caribbean region: Systematic review and narrative synthesis

**DOI:** 10.1371/journal.pone.0282666

**Published:** 2023-03-08

**Authors:** Shaun Liverpool, Yasmin Draoui, Judea Tucker, Brent Pereira, Jamal Prescod, Michael Owen, Catherine Trotman

**Affiliations:** 1 Faculty of Health, Social Care and Medicine, Edge Hill University, Ormskirk, United Kingdom; 2 Evidence Based Practice Unit, Anna Freud National Centre for Children and Families, London, United Kingdom; 3 Department of Counselor Education, The Chicago School of Professional Psychology, Chicago, IL, United States of America; 4 Faculty of Social Sciences, University of the West Indies, Cave Hill, St Michael, Barbados; Access Alliance Multicultural Health and Community Services: Access Alliance, CANADA

## Abstract

**Background:**

Studies conducted in regions consisting of low and middle income and developing countries often report high prevalence of mental health problems among children and young people (CYP). To identify some of the contributing factors we examined the available evidence from research in one such setting.

**Methods:**

Multiple academic databases and grey literature sources were searched until January 2022. We then identified primary research focusing on CYP’s mental health in the English-speaking Caribbean region. Data was extracted and summarized to form a narrative synthesis of the factors associated with CYP’s mental health. The synthesis was then organised according to the social-ecological model. The Joanna Briggs Institute’s critical appraisal tools were used to examine the quality of the reviewed evidence. The study protocol was registered with PROSPERO, CRD42021283161.

**Results:**

From 9684 records, 83 publications representing CYP ages 3 to 24 years from 13 countries met our inclusion criteria. The evidence was varied in quality, quantity and consistency for 21 factors associated with CYP’s mental health. Adverse events and negative peer to peer and sibling relationships were consistently associated with mental health problems, while helpful coping strategies were associated with better mental health. There were mixed findings for age, sex/gender, race/ethnicity, academic level, comorbidity, positive affect, health risks behaviours, religion/prayer, parent history, parent to parent and parent to child relationships, school/employment, geography and social status. There was also some limited evidence for associations between sexuality, screen time and policies/procedures and CYP’s mental health. At least 40% of the evidence contributing to each of the factors was judged as high quality.

**Conclusion:**

Individual, relationship, community and societal factors may influence CYP’s mental health outcomes in the English-speaking Caribbean. Knowledge of these factors is useful to inform early identification and early interventions. More research is needed to explore inconsistent findings and understudied areas.

## Introduction

The mental health and wellbeing of children and young people (CYP) continues to be a global public health concern [1–3]. International evidence suggests that at least 1 in 10 CYP experience symptoms of a mental health problem with 50% of these occurring by age 14 years and 75% by age 24 years [4]. Common internalising and externalising presentations include anxiety, depression and conduct or behaviour problems with high rates of comorbidities among CYP [1, 5]. Notably, some studies highlight disparities in prevalence of mental health problems among CYP identifying as specific minority ethnic groups and those from low and middle income and developing countries [6]. The higher prevalence rates among these groups have mainly been attributed to poverty and social disadvantages but less is known about other risk and protective factors [7, 8]. Although previous reviews suggest some similarities in risk factors for mental health problems in low and middle income and developing countries and those found in high income countries [8, 9], other studies suggest that the factors associated with mental health may be complex and bi-directional and further influenced by culture [10, 11]. Therefore, experts consistently call for more research to provide a deeper understanding of regional differences [12].

The existing literature identified a wide range of demographic, personal, familial, school, social and interpersonal characteristics as key factors associated with CYP’s mental health [13, 14] and subsequent service utilisation [15]. More specifically these factors include age, gender, ethnicity, family composition, urbanisation, family and friend support, social isolation, peer victimization, physical/sexual abuse or emotional neglect and parent psychopathology [16, 17]. In more severe cases additional factors include substance use, comorbid disorders and intellectual disabilities [18, 19]. As for marginalised groups, based on ethnicity or sexuality, experiences of discrimination were also identified as having a negative association with mental health [9, 20]. Conversely, improved self-esteem and optimism have been associated with positive mental health and resilient outcomes [13, 21]. While acknowledging the efforts of researchers in the previous reviews, the evidence from some regions like the English-speaking Caribbean is still under-represented.

The English-speaking Caribbean is made up of about 18 countries or territories, of which the majority are classed as low and middle income or developing status [22–24]. Population statistics suggest that this region consists mainly of families of African, mixed-race, Indian or indigenous origins [25, 26]. Studies conducted in the English-speaking Caribbean region also reported high rates of mental health problems and a limited number of evidence-based interventions [27]. A recent report also highlighted a scarcity of appropriate mental health policies and funding [6]. Due to the complexity of mental health and the great need for services, not all CYP locally or regionally are able to receive professional mental health services [28]. Therefore, there is an obvious demand for a better understanding of the factors that influence CYP’s mental health to develop interventions to identify, prevent and manage mental health problems.

Two previous reviews were conducted in 2009 and 2012 and explored individual as well as micro-and-macro-system factors influencing adolescents’ (10–19-year-olds) mental health in the English-speaking Caribbean region [29, 30]. Between the two reviews the authors identified gender, age, family, home environment, school, religion and engagement in risk behaviours as important factors associated with adolescent’s mental health. Since those studies were conducted there have been an exponential increase in research as well as a growing interest and need for CYP mental health support [27, 31]. There have also been increased attention and advocacy to focus on a wider age range up to 24 years to capture the key transition periods from childhood to adulthood [32]. Therefore, based on recommendations and consensus from academic, practice and lived experience experts, an updated systematic review was deemed appropriate [27, 33].

Subsequently, the aims of the current study were threefold. First, to update the existing reviews to investigate any new or emerging factors that could potentially influence the mental health of CYP in the English-speaking Caribbean region. Second, to build on previous reviews by extending the inclusion criteria to include studies that examined the mental health of CYP below age 10 and up to age 24 years. Third, to improve on the limitations of the previous reviews by conducting quality appraisals of the reviewed articles using established critical appraisal tools.

## Methods

The review process was guided by recommendations from the Joanna Briggs Institute [34], Cochrane Campbell Collaborations [35] and the Preferred Reporting Items for Systematic Reviews and Meta-Analyses (PRISMA checklist, [36]–S1 File). The study protocol was registered with the International Prospective Register of Systematic Reviews (PROSPERO, CRD42021283161).

### Literature search strategy

The first author (SL) conducted the initial literature search in January 2021 and updated the search in January 2022 using academic databases (CINAHL, Cochrane Library, EMBASE, MEDLINE, PsycINFO, LILACS, and Web of Science). We also used grey literature sources (OpenGrey, ResearchGate and the first 10 pages of Google) to track any recent publications that were not yet indexed in the academic databases. Search terms and key words included “children OR adolescent OR young people” AND “mental health OR well-being” AND “West Indies OR Caribbean”. Definitions of mental health and wellbeing were guided by diagnostic manuals (e.g., DSM-5 and ICD 11) and the frame of reference used in the individual studies. The search strategy was developed and piloted through an iterative process with a research librarian. Further details of the literature search have been published as part of the initial scoping review [27].

### Screening and eligibility criteria

Four reviewers (SL, JP, BP, CT) were involved in the two-stage independent screening process. First, titles and abstracts were screened followed by full texts while applying the following criteria. Meetings and email communications were used to resolve disagreements and reach consensus.

Articles were included if:

- Primarily focused on CYP (0–24 years)
- Explored mental health symptoms or diagnoses or any psychosocial problem
- Conducted in the English-speaking Caribbean region
- Detailed peer-reviewed primary research published in English

Articles were excluded if:

- The sample (or over 50%) reported a mean age above 24 years
- The main outcomes were physical health, substance abuse, neurodevelopmental and intellectual disabilities
- Focused on Caribbean migrants or CYP living in non-English-speaking Caribbean countries

### Quality appraisal

The methodological quality of the included studies was assessed using six corresponding checklists from the Joanna Briggs Institute [37]. The checklists assessed methodological quality of the included studies using statements like “Was the sample frame appropriate to address the target population?” and “Was the sample size adequate?”. Fifty-four (65%) of the included studies were independently appraised by three reviewers (JP, SL, BP) resulting in 80% interrater reliability. The remaining studies were appraised by the primary author (SL) and verified by a second reviewer (BP or JP). When necessary, discussions were held to achieve consensus. Items on each checklist were judged as “yes”, “no”, “unclear” or “not applicable”. Similar to other reviews [38, 39], the risk of bias of the contributing results were classified into 1) low risk (high quality evidence), if the studies reached more than 70% of “yes” scores; 2) moderate risk (medium quality evidence), if the studies reached between 50% and 69% of “yes” scores; and 3) high risk (low quality evidence), if the studies reached less than 49% of “yes” scores. Notably some reports presented separate findings from the same dataset. Based on the nature of this review each report was appraised individually, but when necessary, we verified information from the primary study. As this review did not include a meta-analysis the risk of double counting and the related problems were low [40].

### Data extraction

At least two reviewers independently extracted data on publication date, primary author, country of data collection, psychosocial problem explored, study design, sample size, demographics of the sample, outcome measures and recruitment settings. Regarding factor associations, two reviewers (JD and YD) extracted the description of the effect measure between the factors and the outcome, but in cases where no effect measures were present a description of the association was extracted from the text. We also extracted data for negligible and inconsistent associations to provide a non-biased overview of the literature. The information was then verified by a third reviewer (SL or MO) and discrepancies (<10%) discussed at team meetings to reach consensus. The data management was conducted using Microsoft Excel.

### Data analysis

**Fig 1:**
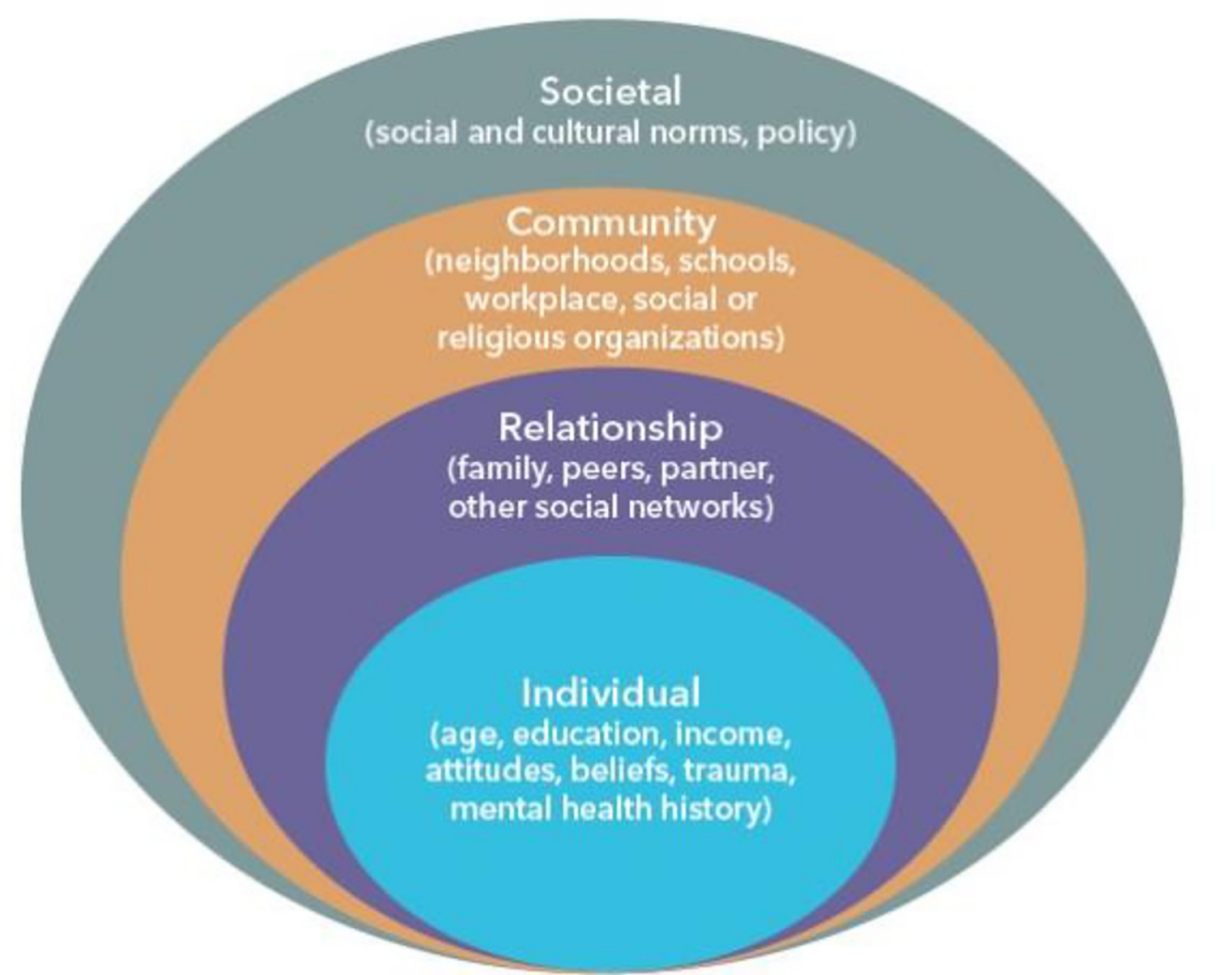
Social ecological model.

First, the study and population characteristics were charted to provide an overall description of the body of evidence. Second, narrative synthesis, supported by thematic and content analysis, as outlined by Popay et al. [41] was conducted. To achieve this, we developed a preliminary synthesis using tables and charts, explored relationships and patterns in the data and then assessed robustness of the synthesis product based on the size, consistency and strength of evidence. Third, factors were then organised into conceptually coherent themes based on the social-ecological model [42, 43]. The social-ecological model was selected as it is a commonly used framework to help understand the complex interplay between individual, relationship, community and societal factors that influence health outcomes (Fig 1).

## Results

**Fig 2:**
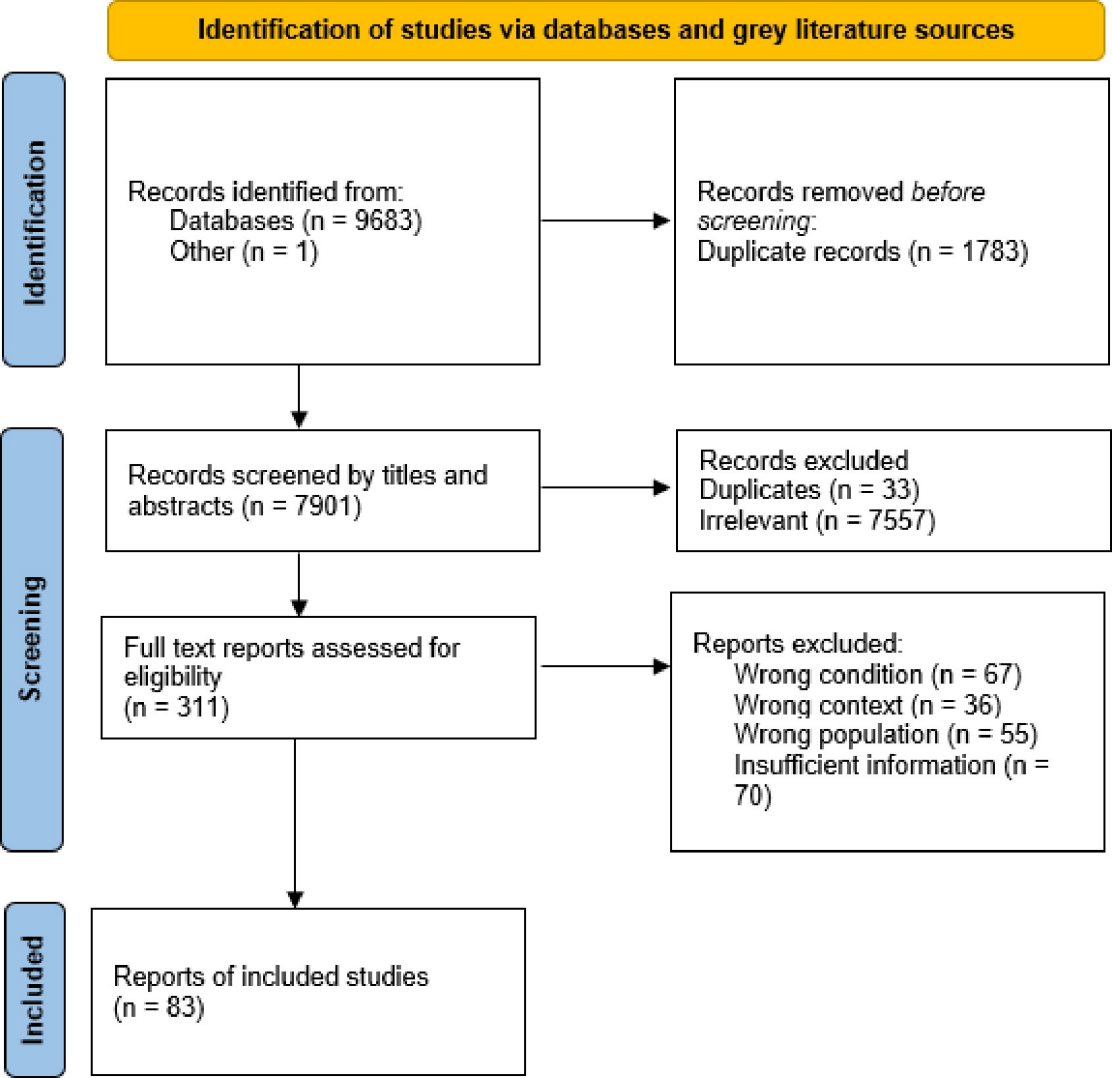
PRISMA flow chart depicting the screening process.

9684 records were retrieved from the database and grey literature searching. 7901 records remained after duplicates were removed. After title and abstract screening, 311 publications were subjected to full-text screening, of which 83 were eligible for inclusion in this review (Fig 2).

### Characteristics of the reviewed studies

The reviewed articles were published between 1976 and 2020 [44–126]. Most of the studies focused on depressive symptoms, behaviour/conduct problems and suicidality. Most of the studies were conducted in Jamaica and Trinidad and Tobago and in education settings. More than half of the reviewed studies were representative in terms of sex (i.e., 50 to 60% males and females) but most focused on adolescents 12 to 17 years. Sample sizes were considerably large with most studies recruiting at least 50 participants. Table 1 provides an overview of the body of evidence and the S2 File provides further details of the reviewed studies.

**Table 1:** Overview of the reviewed articles.

Characteristics of the reviewed articles	Number of articles	% of articles
***Year of publication***		
1976–1989	4	4.82
1990–1999	6	7.23
2000–2009	30	36.14
2010–2020	43	51.81
***Country***		
Jamaica	44	53.01
Trinidad & Tobago	16	19.28
Barbados	5	6.02
Guyana	4	4.82
Bermuda	2	2.41
St Vincent & the Grenadines	1	1.20
The Bahamas	1	1.20
St Kitts & Nevis	1	1.20
St Lucia	1	1.20
Multiple	8	9.64
***Presenting problem***		
Depressive symptoms	25	30.12
Behavioural and conduct problems	17	20.48
Suicidality	15	18.07
Disordered eating & image issues	6	7.23
Anxiety	1	1.20
Multiple	19	22.89
***Recruitment settings***		
Education	66	79.52
Healthcare	12	14.46
Community	5	6.024
***Sex of participants***		
Majority males (>60%)	9	10.84
Majority females (>60%)	24	28.92
50 to 60% males and females	46	55.42
Not clearly stated	4	4.82
***~Age of participants (years)***		
Under 12	9	10.84
12 to 17	46	55.42
18 to 24	28	33.73
***Sample size***		
Small (<50 participants)	12	14.46
Medium (50–300 participants)	25	30.12
Large (>300 participants)	46	55.42

### Factors associated with children and young people’s mental health problems

Twenty-one factors associated with CYP’s mental health were identified and mapped onto the four levels of the social-ecological model. Of the 21 factors, 12 were individual demographic, psychosocial or behavioural factors that included sex/gender, age, comorbidity, academic level, race/ethnicity, sexuality, experience of adverse events, positive affect, health risk behaviours, coping strategies, religion/prayer and screen time. The five relationship factors included parent to parent, parent to child, peer to peer and child to sibling relationships and parent history. The three community factors included school/employment, geography and social status. The only societal factor identified related to existing policies/procedures. Table 2 provides an overview of the factors associated with CYP’s mental health in the English-speaking Caribbean. The blocks are shaded according to the number of studies; darker shading indicates more evidence (number of reports in brackets). The colours represent whether the supporting evidence for each factor association was consistent (green), mixed or inconsistent (yellow) or limited (grey). Factors were judged as having limited or insufficient evidence if they were explored in less than five reports.

**Table 2:** Factors associated with children and young people’s mental health in the English-speaking Caribbean.

INDIVIDUAL			RELATIONSHIP	COMMUNITY	SOCIETY
DEMOGRAPHIC	PSYCHOSOCIAL	BEHAVIOURAL			
**Sex/Gender (43)**	**Adverse events (10)**	**Health risks (14)**	**Parent-parent (18)**	**School/Employment (20)**	**Policies/Procedures (2)**
**Age (21)**	**Positive affect (7)**	**Coping strategies (5)**	**Parent-child (16)**	**Geography (12)**	
**Comorbidity (15)**		**Religion/Prayer (5)**	**Peer-peer (14)**	**Social status (10)**	
**Academic level (8)**		**Screen time (1)**	**Parent history (12)**		
**Race/Ethnicity (8)**			**Child-sibling (5)**		
**Sexuality (1)**					

Note: Darker shading indicates more evidence (number of reports in brackets). Green represents factors with consistent evidence, yellow represents mixed or inconsistent evidence and grey represents limited evidence.

### Quality assessment of the reviewed studies

58 studies (70%) were judged to have low risk of bias, 13 studies (16%) were judged to have moderate risk of bias, and 11 studies (14%) were judged to have high risk of bias. Correspondingly, more studies were of high or medium quality and fewer studies were judged as low quality. The quality assessment details for each study can be found in the S3 File. The quality of the body of evidence informing each factor was organised and presented in Fig 2. Each of the factors comprised of at least 40% of high-quality studies.

### Individual demographic factors

#### 1. Sex/gender

**Fig 3:**
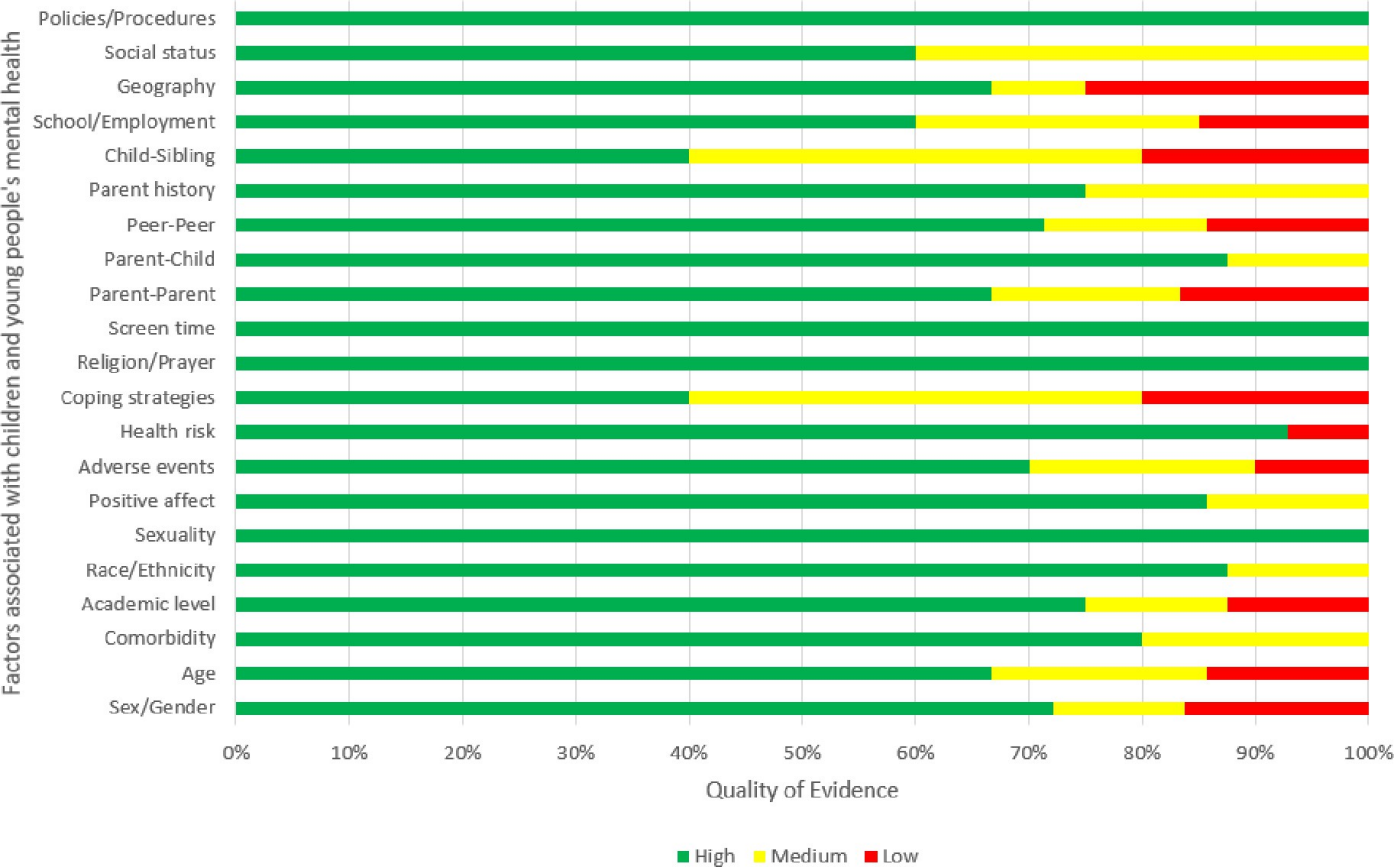
Summary of the quality of evidence for each factor.

There were mixed (or inconsistent) findings on the association between sex/gender and CYP’s mental health. Data from 43 studies informed this association (see Table 2). Of these 31 (72%) were judged as high-quality evidence (see Fig 3).

In a large proportion of the studies (20 out of 43 or 46.5%) females were more likely than males to report depressive symptoms [44–52], disordered eating/body image issues [53–56], psychiatric disorders [57] and other internalising problems [58]. Although females were more likely to have suicide ideation and non-fatal suicide attempts [59–67], males were more likely to be at risk of completing suicide [59, 68]. Similarly, although older females were more likely than males to express significantly higher indirect aggression [69], younger males displayed more verbal and physical aggression [70]. Younger females were also more likely than males to be placed in group homes for behaviour/conduct problems [71], while there were more males than females with mental disabilities in schools [72] and university programmes [73]. Both males and females reported having specific types of phobias [74]. In other studies sex/gender was not associated with depressive symptoms [75–78], suicidal plan or risk [79], stress [80], disordered eating/body image issues [81], aggressive behaviour/conduct problems [82] and other internalising and externalising problems [83–86].

#### 2. Age

There were mixed findings on the association between age and CYP’s mental health. Data from 21 studies informed this association. Of these 14 (67%) were judged as high-quality evidence.

In some studies (6 out of 14 or 42.8%), behaviour/conduct problems and depressive symptoms were more common among older adolescents than primary school-aged children [72] or younger adolescents [50, 51, 76, 82, 87]. However, self-report depressive symptoms did not always increase with age [77]. For example, students who were younger or older than expected for their grade level reported higher depression scores than students who were at the expected age [46]. Younger adolescents were more likely to have suicidal thoughts [62, 63, 67] but more suicide attempt cases were reported among older adolescents and YP (16–20 years) [65]. In terms of behaviour/conduct problems, younger children and adolescents displayed more behaviour/conduct problems than older adolescents [58, 83, 88], but older boys were more likely to be committed for more serious offending behaviours like robbery [71]. Specific phobias and anxiety varied with age; with older students expressing fears of nuclear war and school failure while younger students expressed fears of diseases [74]. In other studies age was not associated with clinical profiles [85], behaviour/conduct problems [89], depressive symptoms [78] or suicidal ideation [66].

#### 3. Comorbidity

There were mixed findings on the association between having a disability and CYP’s mental health. Data from 15 studies informed this association. Of these 12 (80%) were judged as high-quality evidence.

Most studies (14 out of 15 or 93.3%) suggested that the presence of mental health symptoms was common when YP experienced other chronic conditions or disabilities. For example, studies suggested that having learning difficulties was associated with more depressive symptoms [47], behaviour/conduct problems and anxiety [90]. Studies also suggested that having chronic and acute mental and physical conditions, including self-harm practices, were associated with disordered eating attitudes [54, 81], depressive symptoms [91], behaviour/conduct problems [85, 89], suicide ideation [60, 63, 66, 67, 92, 93] and other psychiatric disorders [57]. Notably, one study suggested that mental disorders was not frequently found in YP with behaviour/conduct problems [94].

#### 4. Academic level

There were consistent findings on the association between level of education or academic performance and CYP’s mental health. Data from 8 studies informed this association. Of these 6 (75%) were judged as high-quality evidence.

Studies suggested that YP with higher levels of education or better academic performance experienced lower depressive symptoms [48, 95], were less likely to plan suicide [64] and were at reduced risk of anxiety [90]. In terms of behaviour/conduct problems, inadequate education or poorer academic performance was associated with increased behaviour/conduct problems [89, 94], but higher grade levels were also sometimes significantly associated with more frequent behaviour/conduct problems [87]. Among older students, being in the final year of university or those with academic issues were more likely than other students to experience burnout, higher stress levels and other mental disorders [95, 96].

#### 5. Race/Ethnicity

There were mixed findings on the association between race/ethnicity and CYP’s mental health problems. Data from 8 studies informed this association. Of these 7 (88%) were judged as high-quality evidence.

Mixed-race YP (i.e., African and Indian ethnic origins) and Afro-Trinidadians were more likely than Indo-Trinidadians to report higher levels of suicidality [64], and in Guyana, callers to a suicide hotline were more likely to be Indo-Guyanese [59]. Regarding disordered eating/body image issues, one study suggested Indo-Trinidadians were more likely than Afro-Trinidadians and mixed-race Trinidadians to report body dissatisfaction/eating issues [97] contradicting another study which suggested that Afro-Trinidadian adolescent females reported significantly higher scores on body dissatisfaction and binge eating practices [98]. Relatedly, a desire for lighter skin complexion was also associated with disordered eating/body image issues [54]. Regarding behaviour/conduct problems, more Afro-Trinidadians than other ethnicities were represented at group homes [71]. In other studies race/ethnicity was not significantly associated with suicidality [61] or depressive symptoms [44].

#### 6. Sexuality

One high quality study suggested that YP with conflict surrounding their sexual orientation were more likely to experience emotional and social distress [59].

### Individual psychosocial factors

#### 7. Positive affect

There were mixed findings on the association between positive affect and CYP’s mental health. Data from 7 studies informed this association. Of these 6 (86%) were judged as high-quality evidence.

In most studies (5 out of 7 or 71.4%), increased self-esteem, life satisfaction, resilience and positive emotions (e.g., happiness) was associated with lower levels of depression, anxiety, stress, aggressive behaviours and increased psychological wellbeing [99–103]. As for disordered eating, this association was mediated by gender, indicating that females with higher self-esteem were at reduced risk of disordered eating but this association was not significant for males [54]. However, in one study there was no significant association between self-esteem and depressive symptoms [50].

#### 8. Adverse events

There were consistent findings on the association between negative affect or adverse experiences and CYP’s mental health. Data from 10 studies informed this association. Of these 7 (70%) were judged as high-quality evidence.

All of these studies (10 out of 10, 100%) suggested that YP who were lonely, unhappy, traumatised from experiences of abuse (e.g., sexual, verbal and physical) or had thoughts of harming self or others or decreased life expectancy or lower future ambitions, or negative opinions and thoughts were more likely to report higher levels of distress [104], depressive symptoms [50, 86], suicidal ideation and attempts [62, 63, 66, 67, 105] and behaviour/conduct problems [66, 94, 106].

### Individual behavioural factors

#### 9. Health risk behaviours

There were mixed findings on the association between health risk behaviours and CYP’s mental health. Data from 14 studies informed this association. Of these 13 (93%) were judged as high-quality evidence.

Most studies (13 out 14 or 92.9%) suggested that YP who admitted to alcohol or drug (mis)use (e.g., cannabis) and unsafe sexual practices were at increased risk of disordered eating/body image issues [54, 81], psychotic and depressive symptoms [45, 77, 107, 108] and behaviour/conduct problems [87, 109]. However, there were mixed findings for suicide ideation indicating that alcohol or drug (mis)use was associated with suicide ideation [60, 62, 63, 67, 89] but the association was not always significant [79].

#### 10. Coping strategies

There were consistent findings on the association between self-care strategies and CYP’s mental health. Data from 5 studies informed this association. Of these 2 (40%) were judged as high-quality evidence.

Avoidant, emotional and support coping were used by YP to manage problem behaviours [86, 110] and relaxation techniques like reading, deep breathing and sleeping were used to manage stress [111]. These techniques alongside being in control of their daily schedules were associated with reduced rates of burnout and depressive symptoms in older YP [95]. Effective social adjustment skills were also associated with reduced psychiatric distress [57].

#### 11. Religion/Prayer

There were mixed findings on the association between religion or prayer and CYP’s mental health. Data from 5 studies informed this association. All studies were judged as high-quality evidence.

All of the studies (5 out of 5 or 100%) suggested that YP who identified as having a religion (e.g., Catholic, Seventh-day Adventists and Pentecostal) or those who attended a place of worship were less likely than YP with no religious affiliation to have suicide ideation/attempts [61, 64] or behaviour/conduct problems [89] or burnout and depressive symptoms [44, 95]. However, one study suggested that YP identifying as non-Christian religions were at increased risk of suicide ideation/attempts [64]. Notwithstanding statistical significance the same study found that YP who reported praying with their families were less likely to experience suicide ideation/attempt [64].

#### 12. Screen time

One high quality study suggested that there was no significant difference between the amount of television watched and aggressive or prosocial behaviours in CYP [89].

### Relationship factors

#### 13. Parent to parent

There were mixed findings on the association between parent-to-parent relationship (e.g., parental conflict or separation) and CYP’s mental health. Data from 18 studies informed this association. Of these 12 (67%) were judged as high-quality evidence.

In most of the studies (14 out of 18 or 77.8%) CYP living with unmarried parents, parents in conflict, or reconstructed families (e.g., living with relatives) were more likely than other groups to report symptoms of mental health problems. For example, CYP living with unmarried parents or reconstructed families were more likely than other groups to report disordered eating/body image issues [53, 54, 81], depressive symptoms [44, 50, 112] or suicide ideation [61, 86]. Similarly, YP not living with their both parents or experiencing other sources of family conflict were at increased risk of disordered eating/body image issues [54, 77], depressive symptoms [45, 96], stress [57, 59] and behaviour/conduct problems [113]. In terms of behaviour/conduct problems, some evidence suggested the absence of a parent or separation did not have a significant impact if the separation occurred within the first five years of the child’s life, while separation later in the young person’s life was associated with psychological distress [94, 112]. Reasons for parental separation, for example, witnessing inter-adult verbal aggression or domestic violence also predicted behaviour/conduct problems [89], depressive symptoms, suicide ideation and psychological distress in YP [82]. In another study, family conflict was not significantly associated with YP’s mental health [114].

#### 14. Parent to child

There were mixed findings on the association between parent to child relationships and CYP’s mental health. Data from 16 studies informed this association. Of these 14 (88%) were judged as high-quality evidence.

YP who reported being afraid of their parents or had unhealthy attachments to their mothers were more likely than others to report depressive symptoms [45], but YP with stronger attachments to their mothers were also likely to display offending behaviours like owning a gun [114]. Authoritarian or neglectful parenting styles and using physical punishment or excess monitoring also increased the risk of YP experiencing psychological maladjustment [115], behaviour/conduct problems [89], depressive symptoms [77, 116], or suicide ideation [70], but an agreement between YP and parents on the style of parenting reduced the risk of poor psychosocial outcomes [69, 70]. The reverse occurred in other studies with parental monitoring of free time being associated with lower odds of mental health problems [66, 117]. However, one study reported that the severity of parental punishment alone had little effect on the variations in YPs psychological adjustments [115]. One study also found no significant association between corporal punishment and psychosocial outcomes, but a significant negative association between parent-child verbal punishment and psychological outcomes [82].

Conversely, YP who described their parents as understanding and YP who received regular emotional or social support from their family were at reduced risk of burnout, depressive symptoms or suicide ideation [62, 63, 99, 105] and overall psychological distress [101]. Yet, one study found that some YP who had parental understanding were still more likely than other YP to experience suicidality [67].

#### 15. Peer to peer

There were consistent findings on the association between peer relationships and CYP’s mental health. Data from 14 studies informed this association. Of these 10 (71%) were judged as high-quality evidence.

YP who experienced peer pressure were more likely to feel overwhelmed and in need of emotional support [59]. Similarly, YP who were victims of bullying were also more likely to experience suicide ideation [60, 63, 84] and excessive worry [84]. However, YP with close friendships, good interpersonal skills and in receipt of social support were less likely to experience suicidal thoughts [60, 62, 63, 93] and reduced psychological wellbeing [99, 101]. In terms of behaviour/conduct problems, YP with friends who were in trouble with the law were more likely than other YP to display delinquent behaviours [71].

Studies also suggested that YP who were not in committed relationships or YP who experienced conflict in their romantic relationships were more likely than other YP to experience suicidal thoughts [59, 65], psychological distress [57, 100] or stress and anxiety [96]. Among older YP, married students reported significantly lower depressive symptoms than students in visiting relationships [47].

#### 16. Parent history

There were mixed findings on the association between parent history or background and CYP’s mental health. Data from 12 studies informed this association. Of these 9 (75%) were judged as high-quality evidence.

In 50% of these studies (6 out of 12 studies) YP with family members with mental health problems, legal issues or substance (mis)use were at increased risk of disordered eating/body image issues [81], depressive symptoms [44], behaviour/conduct problems [71, 89, 114] and suicidality [61]. This also meant that when parents exhibited tolerant attitudes towards drug use and gun ownership YP were more likely to display offending behaviour/conduct problems [113, 114]. One study noted however that mental disorders and criminal activity was infrequently found among parents of YP with delinquent behaviours [94] but poor family management and lack of specific practices (e.g., structured mealtimes) was associated with emotional distress in YP [114, 118]. As for parent education level, YP whose mothers had post-secondary education were at reduced risk of depressive symptoms [46–48]. However, one study suggested that there was no significant association between parent education and behaviour/conduct problems in YP [89].

#### 17. Child to sibling

There were consistent findings on the association between relationships among siblings and CYP’s mental health. Data from 5 studies informed this association. Of these 2 (40%) were judged as high-quality evidence.

As for other family relationships, YP with male siblings or multiple siblings or siblings with a history of antisocial behaviours were significantly more likely than other YP to display specific behaviour/conduct problems like gun ownership [94, 113, 114] or be at increased risk of disordered eating/body image issues [81]. YP with a chronically ill sibling also reported greater distress and poorer social adjustments than other YP [119].

### Community factors

#### 18. School/Employment

There were mixed findings on the association between school/university environment, employment and CYP’s mental health. Data from 20 studies informed this association. Of these 12 (60%) were judged as high-quality evidence.

YP attending non-traditional and non-prestigious high schools were more likely than other students to report depressive symptoms [44, 75]. However, no significant association was found between the type of school or sense of belonging and behaviour/conduct problems [87] and suicidal ideation [61] in other studies. Studies also suggested an association between missing school or classes and suicidality [62, 63] and behaviour/conduct behaviours [120]. Relationships with teachers, sometimes impacted by school punishments (e.g., beaten by hand) was inversely associated with behaviour/conduct problems [87, 89, 110, 121]. Among older YP (e.g., university students) the amount of material to be studied, exams/grades, campus facilities and quality of teaching contributed to stress levels [80, 100]; while combining employment and studies resulted in lower occurrences of depressive symptoms [47] or psychiatric disorders [57]. Specifically, students attending nursing programmes experienced moderately high levels of stress in clinical environments [111]. Notably, in one study students were also more likely than non-students to report suicidality [68]. As for the school environment, one study suggested that schools offering mental health interventions (e.g., whole school approaches) were not significantly beneficial to YP’s mental health [121], but other studies suggested some improvements in behaviour problems like the use of profanity [122, 123]. Another study highlighted that a universal intervention in a school was useful in supporting children with behaviour and conduct problems but not for prosocial and emotional problems [124]. However, another multi-modal intervention implemented in schools made significant improvement in school social and behaviour adjustments, particularly for boys [102].

#### 19. Geography

There were mixed findings on the association between geographic location and CYP’s mental health problems. Data from 12 studies informed this association. Of these 8 (67%) were judged as high-quality evidence.

YP from rural areas were more likely than other YP to display externalising problems [83] and suicidality [68]. In other studies, YP from urban communities or violent prone areas were more likely that other YP to report suicidal behaviours [79], depressive symptoms [50], disordered eating [56] and offending behaviours [94]. On a country level, one study suggested that YP from Jamaica reported significantly higher depressive symptoms than YP from St Kitts and Nevis or St Vincent and the Grenadines [48, 116], while another study suggested that YP from the Bahamas had higher levels of depressive symptoms than YP from Jamaica [125]. Community opportunities and rewards for prosocial involvement did not have statistically significant association with behaviour/conduct problems [109] but socially organised communities or higher quality neighbourhoods (e.g., lower crime rates) were more likely to contribute to reduced behaviour/conduct problems [89] and depressive symptoms [109, 125, 126]. Having a sense of belonging to a particular neighbourhood was also associated with lower levels of depressive symptoms [125, 126].

#### 20. Social status

There were mixed findings on the association between social status and CYP’s mental health. Data from 10 studies informed this association. Of these 6 (60%) were judged as high-quality evidence.

In most of the studies (6 out of 10 studies, 60%) YP with parents who were unemployed or underemployed or had manual jobs (e.g., plumbing) or those who belonged to poorer households were at increased risk of disordered eating [81], depressive symptoms [51] or behaviour/conduct problems [58, 71, 89, 94]. This also meant that for females the lack of domestic amenities (e.g., water) or YP who were undernourished had increased psychological distress [54, 76]. For example, even YP from high economic status families who were unable to have time for family meals or those with financial difficulties resulting in lack of food security were more likely to report psychological distress [63, 111].

### Societal factors

#### 21. Policies or procedures

There were mixed findings on the association between policies/procedures and CYP’s mental health. Data from 2 studies informed this association. Both studies were judged as high-quality evidence.

Although laws and norms disfavouring drug use and firearms did not have a significant effect on offending behaviours, the risk of apprehension was significantly associated with less frequent gang membership [109]. Other institutional policies, procedures, and regional norms also sometimes contributed to contention and disappointment among CYP [101].

## Discussion

### Summary of findings

Our systematic review and analysis of 83 reports identified 21 factors with varied evidence of associations with CYP’s mental health across four levels (i.e., individual, relationship, community and society). These factors include age, sex/gender, race/ethnicity, academic level, comorbidities, sexuality, positive affect, adverse events, health risk behaviours, coping strategies, religion/prayer, screen time, parent to parent, parent to child, peer to peer and child to sibling relationships, parent history, social status, school/employment, geography and policies/procedures.

### Comparison to previous reviews

In line with previous reviews, our study confirms the association of individual, relationship, community and societal factors with the mental health of CYP [13–21, 29]. Unlike previous reviews there were mixed finding for some of the key factors like parent-to-parent relationship and social status. This further adds to the body of evidence suggesting that mental health is complex and not all CYP may be affected in the same way by the various factors [10, 11]. Similar to other reviews sexuality and screen time were explored in very few studies and resulted in limited evidence on which to draw stronger conclusions. In addition to the factors identified in the international literature, we found religion/prayer and policies/procedures to be associated with mental health outcomes in CYP in the English-speaking Caribbean.

We also built on the two regional reviews by identifying child to sibling relationships, coping strategies, screen time, sexuality and policies/procedures as emerging factors that have not been previously explored. One possible explanation for any of the differences could be methodological. For example, our review included a wider age range (3–24 years) compared to other reviews, allowing us to capture issues around romantic relationships and sexual experiences under individual and relationship factors as possible sources of distress for CYP. These factors appeared to be missed or understudied in some of the international literature around CYP under age 18. Our findings further highlight the importance of exploring these factors, especially in regions like the English-speaking Caribbean where higher rates of teenage pregnancies and risky sexual behaviours are common [30, 127].

The second possible explanation could be cultural. Notwithstanding the mixed findings, CYP’s identification with religion/prayer was seen as potential to be a protective factor against poorer mental health outcomes. The mixed findings could suggest that a deeper understanding of the differences between spiritual views and religious affiliations among CYP would be needed before moving forward. This is especially important as some CYP and their families seek out mental health support from religious leaders [128].

Lastly, similar to other reviews, the studies included in our review were of varying quality. However, in line with recommendations for a narrative synthesis, we also explored the body of evidence contributing to each factor allowing us to make specific recommendations with greater confidence [41]. Despite urgent calls for mental health support for CYP in the Caribbean [31], the amount of research that investigated some of the influencing factors (e.g., screen time and sexuality) appears to be small relative to other regions [20, 129].

### Implications for future research

Owing to the mixed findings on some of the associating factors (e.g., parent to child relationships) and the dearth of evidence on others (e.g., sexuality), we suggest that further research is needed to better our understanding as to why and in what context some CYP are at greater risk of developing mental health problems. Further cross-disciplinary research focusing on possible interactions among these factors would also be useful since the evidence reviewed did not always provide a consistent narrative of the risk profiles. The absence of some key factors (e.g., impact of natural disasters), presenting problems (e.g., psychosis), and countries (e.g., Belize) also reinforces recent appeals for more research in this area [27]. Despite the gradual increase in research activity in the last 40 years, there was also a lack of diversity in research methods used in the included studies and variation in the quality of the evidence, suggesting a need for a wider range of high-quality rigorous research activity.

### Implications for policy

To our knowledge, this review of 83 articles is the largest and most up to date review of the evidence on the factors that contribute to psychological distress among CYP in the English-speaking Caribbean which comprise of majority low-and-middle-income and developing countries. Therefore, policy makers can use the findings as a guide during key decision-making periods. Also, based on the findings in this review the impact of policies/procedures on CYP is not yet clear. However, it appears that in some instances policies can be a source of distress for CYP; so, it is recommended that CYP are involved in the development of policies that are relevant to them [130].

### Implications for practice

This review identified several factors that have the potential to overlap or influence other factors. Knowledge of these factors can help professionals identify CYP at risk of developing psychosocial problems or at risk of experiencing worsening mental health symptoms. Our findings also suggest a need for early interventions that can support CYP at different stages and in different settings. There are also opportunities to promote protective factors like helpful coping strategies and positive affect. By focusing on factors across the various levels there is also potential to achieve population impact. For example, universal interventions focusing on schools and families or those targeting specific groups of CYP based on sex/gender or religious affiliations may be beneficial. Notably, emerging evidence from the Caribbean suggest an interest for innovative delivery methods for CYP to receive support [131]. Therefore, these could include mobile apps and online resources or a combination of different approaches.

### Strengths and limitations

The main strength of this review is the conceptual organisation of factors associated with CYP’s mental health which contributes to the theoretical framework for identifying CYP who might be most at risk of mental health problems. The other strengths of this study include our comprehensive search strategy applied to academic databases and grey literature sources and the inclusion of peer reviewed articles. This study also benefited from the participation of at least two reviewers during screening and study selection, quality appraisal, data extraction and the narrative synthesis which could strengthen the reliability of our findings. However, the review is not devoid of limitations. Despite our best attempts to identify all relevant studies some information could have been missed. It is also important to note that this review pooled data from a number of studies, each with its own limitations. The decision to only include peer-reviewed material could also contribute to publication bias. Another limitation is the underrepresentation of specific age groups (e.g., <12 years), psychosocial problems (e.g., eating disorders), geographic locations (e.g., Grenada) and limited exploration of some common associating factors (e.g., sexuality). However, where appropriate the authors were explicit in reporting specific information; but in keeping with guidelines for the narrative synthesis this was not always possible. Therefore, caution is advised if attempts are made to generalize our findings.

## Conclusions

It is well established that CYP from regions consisting of low and middle income and developing countries are at increased risk of experiencing mental health problems. This review adds to the international evidence by providing insights on 21 factors associated with CYP’s mental health in one such region. Of these, the role of religion/prayer had not been fully explored in the international reviews. Sexuality, screentime and the impact of policies/procedures are understudied and other factors like gender issues and wider societal problems (e.g., Covid-19) are not yet explored. Our findings support the relevance of considering individual, relationship, community and societal aspects in early identification and early interventions aimed at CYP’s mental health and wellbeing.

## Supporting information

S1 File PRISMA checklist. (DOCX)

S2 File Characteristics of the included studies. (PDF)

S3 File Quality appraisal. (XLSX)

## Abbreviations

CYP: Children and young people
YP: Young people
