# Supplementary material for: Factors associated with children and young people’s mental health in the English-speaking Caribbean region: Systematic review and narrative synthesis
Source: PLoS One. 2023 Mar 8;18(3):e0282666. doi: 10.1371/journal.pone.0282666 (PMC9994705; doi:10.1371/journal.pone.0282666)
Supplement: S2 File — (PDF) [file pone.0282666.s002.pdf]

Supplementary Information 2. Characteristics of the reviewed studies (K=83)

Reference: First author, year of publication	Country	Presenting problem explored	Outcome measures	Study design	Sample size	MeanAge (SD) or age range in years	Predominant sex (%)	Primary recruitment setting
Harrison, 2020	Jamaica	Disordered Eating Behaviours and Attitudes	Eating Attitude Test (EAT); Rosenberg Self- Esteem (RSE) scale	Quantitative	521	14.8 (not specified)	56.05% females	Education
Chung, 2020	Jamaica	Depressive symptoms	The Adolescent Depression Rating Scale (ADRS)	Quantitative	321	10 to 12	49.8% females (7.8% did not respond)	Education
Johnson, 2019	Guyana	Suicidality	Not applicable	Qualitative	13	Adolescents	55.41% females	Community
Gardner, 2019	Jamaica	Multiple: self-esteem, psychological well-being, stress, anxiety, and depression	Depression, Anxiety, and Stress Scale–Short Form (DASS- 21); Psychological Well-being Scale–Short Version (PWB-18); Multidimensional Scale of Perceived Social Support; Rosenberg's Self-Esteem (RSE) Scale	Quantitative	334	14.56 (1.94)	51% males	Education
Baker-Henningham, 2019	Jamaica	Behaviour and conduct problems	Observations; Emotional Support Domain in the Classroom Assessment Scoring System (CLASS-K- 3)	Quantitative	220	6 to 7	54% males	Education
Elledge, 2019	Jamaica	Multiple: suicidality, internal distress (worry, loneliness)	Jamaica Global School- based Health Survey (GSHS)	Quantitative	1623	11 to 16	51.2% females	Education
Bateman, 2019	Jamaica	PTSD	Impact of Event Scale- Revised (IES-R); General Self-Efficacy Scale (GSES)	Quantitative	46	18 to 24	65.2% males	Education
Gardner, 2019	Jamaica	Depressive symptoms	Depression, Anxiety and Stress Scale Short (DASS) Form; Rosenberg Self- esteem (RSE) Scale; Schutte Emotional Intelligence Scale	Quantitative	334	14.74 (1.95)	51% males	Education
Descartes, 2019	Trinidad and Tobago	Behaviour and conduct problems	Direct and Indirect Aggression Scale (DIAS); Culture Free Self-Esteem Inventories (CFSEI-3)	Quantitative	170	9 to 15	52% females	Education
Oshi, 2018	Barbados	Behaviour and conduct problems	National Secondary Schools Survey	Quantitative	8109	14 (not specified)	59% females	Education
Debowska, 2018	Barbados & Grenada	Behaviour and conduct problems	Author developed/adapted questionnaire	Quantitative	1351	9 to 17	50.1% females	Education
Giray, 2018	Jamaica	Depressive symptoms	Jamaican Youth Risk and Resiliency Behaviour Survey, Patient Health Questionnaire (Adapted)	Quantitative	366	13.8 (not specified)	64% females	Education
Heron, 2017	Jamaica	Suicidality	Suicidal Behaviours Questionnaire-Revised (SBQ_R)	Quantitative	3471	10 to 19	58.8% females	Education
Kwangu, 2017	Bahamas	Suicidality	Global School-based student Health Survey (GSHS)	Quantitative	1357	13 to 17	53.1% females	Education

Siziya, 2017	Guyana	Suicidality	Global School-based Health Survey (GSHS)	Quantitative	2392	Mode 14 to 15	51.5% female	Education
Siziya, 2017	Jamaica	Suicidality	Jamaica Global School- based Health Survey (GSHS)	Quantitative	1623	Grade 7 to 12	51.4% males	Education
Denton, 2017	Guyana	Suicidality	DSM-5 Level 1 Cross- Cutting Symptom Measure; Behavioural Assessment Schedule for Children, 2nd Edition (BASC-2)	Mixed Methods	25	12.7 (not specified)	52% females	Healthcare
Graham, 2016	Jamaica	Stress	Author developed/adapted questionnaire	Quantitative	106	23.16 (9.0)	97.2% females	Education
Youssef, 2016	Trinidad and Tobago	Multiple: depressive symptoms and stress	Maslach Burnout Inventory (MBI); Perceived Medical School Stress scale (PMSS); Patient Health Questionnaire (PHQ-9)	Quantitative	381	22.4 (3)	67% females	Education
Maguire, 2016	Trinidad and Tobago	Behaviour and conduct problems	Trinidad & Tobago Youth Survey (TTYS)	Quantitative	2552	15.4 (not specified)	60% females	Education
Harrison, 2015	Jamaica	Disordered Eating Behaviours and Attitudes	Rosenberg Self-Esteem (RSE) scale; EAT-26 scale; Parent and Peer Attachment Scales.	Quantitative	524	14.9 (not specified)	56% females	Education
Toussaint, 2015	Trinidad and Tobago	Suicidality	Trend Research Empowering National Development (TREND)	Quantitative	4448	18.14 (1.16)	57% females	Community
Lowe, 2014	Jamaica, Bahamas, St. Vincent and the Grenadines & St. Kitts & Nevis	Depressive symptoms	Beck Depression Inventory (BDI-II)	Quantitative	1955	15.3 (0.95)	52.1% females	Education
Wilson-Mitchel, 2014	Jamaica	Multiple: psychological distress or suicidality	Not applicable	Mixed Methods	30	15.6 (0.93)	100% females	Healthcare
McFarlane, 2014	Jamaica	Depressive symptoms	Jamaica Youth Risk and Resiliency Behaviour Survey; The Ministry of Health screening tool (adaptation of the Diagnostic and Statistical Manual of Mental Disorders 4th Edition)	Quantitative	1312	15 to 19	54.57% females	Community
Maguire, 2013	Trinidad and Tobago	Behaviour and conduct problems	Trinidad and Tobago Youth Survey (TTYS)	Quantitative	2552	15.4 (not specified)	60% females	Education
Smith, 2013	Jamaica	Multiple: psychological and behavioural wellbeing	Massachusetts Youth Screening Instrument: Version 2 (MAYSI-2)	Quantitative	563	15.10 (not specified)	57.2% females	Education
Guzder, 2013	Jamaica	Behaviour and conduct problems	Achenbach System of Empirically Based Assessment (ASEBA) Teacher Report Form	Quantitative	30	9.1 (0.41)	57% males	Education

Smith, 2013	Jamaica	Multiple	Massachusetts Youth Screening Instrument Version 2 (MAYSI-2); Author-developed behaviour problem questionnaire.	Quantitative	171	15.14 (1.40)	54.2% males	Education
Baker-Henningham, 2012	Jamaica	Behaviour and conduct problems	Not applicable	Quantitative	225	3 to 6	61% males	Education
Lipps, 2012	Jamaica, Bahamas, St. Vincent and the Grenadines & St. Kitts and Nevis	Depressive symptoms	Parenting Practices Scales (PPS); Beck Depression Inventory - II (BDI-II)	Quantitative	1955	15.3 (0.95)	52.1% females	Education
Abdirahman, 2012	Cayman Islands, St Lucia, St Vincent and the Grenadines, and Trinidad and Tobago	Multiple: depression, anxiety, loneliness	Global School-based Student Health Survey (GSHS)	Quantitative	6780	13 to 15	52.98% females	Education
Abel, 2012	Jamaica	Depressive symptoms	Caribbean Adolescent Health Survey (PAHO); Global School Health Survey (WHO),	Quantitative	3003	12.45 (1.68)	52.65 % females	Education
Holder-Nevins, 2012	Jamaica	Suicidality	Case notes	Quantitative	26	16 (3.01)	76.9% males	Community Police
Williams-Johnson, 2012	Jamaica	Suicidality	Author developed/adapted questionnaire	Quantitative	127	Mode 16 to 20 and 56.7% under 25	75.6% females	Healthcare
Abell, 2012	Jamaica	Suicidality	Global School Health Survey (WHO)	Quantitative	2997	10 to 15	52.69% females	Education
Maguire, 2011	Trinidad and Tobago	Behaviour and conduct problems	Trinidad and Tobago Youth Survey (TTYS)	Quantitative	2552	15.4 (not specified)	60% females	Education
Smith, 2011	Jamaica	Multiple	Massachusetts Youth Screening Instrument-2 (MAYSI-2); Rosenberg Self-esteem Scale	Quantitative	563	15.08 (not specified)	57.2% females	Education
Lipps, 2010	Jamaica	Depressive symptoms	Beck Depression Inventory–II (BDI-II)	Quantitative	278	15.0 (0.6)	52% females	Education
Galler, 2010	Barbados	Depressive symptoms	Minnesota General Adjustment and Morale Scale	Quantitative	177	11 to 17	Unclear	Education
Alleyne, 2010	Barbados	Multiple: stress and life satisfaction	Satisfaction with Life Scale (SLS); Perceived Stress Scale (PSS)	Quantitative	172	24.9 (not specified)	70% females	Education

Kukoyi, 2010	Jamaica	Suicidality	9-country Caribbean survey (Adapted)	Quantitative	342	10 to 19	52.2% females	Education
Lipps, 2010	Jamaica, St. Vincent and the Grenadines & St. Kitts and Nevis	Depressive symptoms	Beck Depression Inventory II (BDI-II)	Quantitative	1738	12 to 19	52% females	Education
Maynard, 2009	Barbados	Behaviour and conduct problems	Not applicable	Qualitative	20	14 to 16	50% males	Education
Lowe, 2009	St. Kitts & Nevis	Depressive symptoms	Beck Depression Inventory- II (BDI-II)	Quantitative	744	15.5 (0.8)	50.4% females	Education
Lowe, 2009	Jamaica	Depressive symptoms	Brief Screen for Depression (BSD: 16)	Quantitative	690	23.4 (7.4)	77% females	Education
Baker-Henningham, 2009	Jamaica	Behaviour and conduct problems	Strengths and Difficulties Questionnaire (SDQ)	Quantitative	135	Pre-schoolers	65.19% males	Education
Nichols, 2009	Barbados, Jamaica, Trinidad and Tobago	Multiple: disordered eating, depression	Eating Attitudes Test (EAT-26); Body Shape Questionnaire (BSQ-16); Body Silhouette Chart; Rosenberg Self-Esteem Scale (RSE); Centre for Epidemiologic Studies depression scale (CES-D)	Quantitative	383	21 (2.8)	51.96 males	Education
Maharaj, 2008	Trinidad and Tobago	Depressive symptoms	Beck Depression Inventory (BDI-II)	Quantitative	1290	13 to 19	58.2% females	Education
Turton, 2007	Bermuda	Behaviour and conduct problems	Not applicable	Qualitative	1	16-year-old	100% females	Education
Rudatsikira, 2007	Guyana	Suicidality	Guyana Global School- Based HealthSurvey (GSHS)	Quantitative	1197	14 (not specified)	51% females	Education
Ekundayo, 2007	Jamaica	Depressive symptoms	Beck's Depression inventory II (BDI-II)	Quantitative	748	14 to 19	64.7% females	Education
Meeks Gardner, 2007	Jamaica	Behaviour and conduct problems	Author developed/adapted questionnaire	Quantitative	202	11.6 (0.6)	100% males	Education
Lipps, 2007	Jamaica	Depressive symptoms	Beck Depression Inventory–II (BDI-II); Centre for Epidemiology Studies-Depression (CES- D); Brief Screen for Depression (BSD); University of Los Angelos Loneliness Scale-Revised (UCLA-R)	Quantitative	690	23.4 (7.4)	77% females	Education
Maharajh, 2006	Trinidad and Tobago	Depressive symptoms	Reynolds Adolescent Depression Scale (RADS)	Quantitative	1845	16.3 (1.13)	60% females	Education

Steely, 2006	Jamaica	Multiple: psychological adjustment	Family History Assessment Form; Physical Punishment Questionnaire: Youth Form (Youth PPQ); Parental Acceptance- Rejection Questionnaire/Short Form (PARQ/Short Form); Personality Assessment Questionnaire: Child Version (Child PAQ)	Quantitative	97	7 to 18	55% females	Healthcare
Ramberan, 2006	Trinidad and Tobago	Disordered Eating Behaviours and Attitudes	EATing Attitudes Test (EATS-26); Body Shape Questionnaire (BSQ-16); Body Silhouette Chart; Rosenberg Self-Esteem Scale (RSE); Drive for thinness subscale of the Eating Disorder Inventory 2.	Quantitative	251	16.3 (1.37)	100% females	Education
Lipps, 2006	Jamaica	Depressive symptoms	Brief Screen for Depression (BSD); Beck Depression Inventory (BDI-II); Centre for Epidemiology Studies- Depression (CES-D); University of Los Angelos Loneliness Scale-Revised (UCLA-R)	Quantitative	244	22.7 (not specified)	81.97% females	Education
Marlowe, 2005	Bermuda	Disordered Eating Behaviours and Attitudes	Bulimic Investigatory Test, Edinburgh (BITE); Eating Attitudes Test (EAT-40)	Quantitative	836	12.2 (0.66)	52.5% females	Education
Maharajh, 2005	Trinidad and Tobago	Suicidality	Community Assessment of Psychic Experiences (CAPE) questionnaire	Quantitative	227	12 to 20	Unclear	Education
Pottinger, 2005	Jamaica	Loss and grief	Author developed/adapted questionnaire	Quantitative	54	9 to 10	52% males	Education
Maharajh, 2005	Trinidad and Tobago	Multiple	Case notes (DSM criteria)	Quantitative	349	14.4(2.85)	60% males	Healthcare
Ali, 2005	Trinidad and Tobago	Suicidality	The Suicidal Ideation Questionnaire (SIQ)	Quantitative	1845	14 to 20	60% females	Education
Maharajh, 2004	Trinidad and Tobago	Depressive symptoms	Reynolds Adolescent Depression Scale (RADS); Patient Health Questionnaires (PHQ-9)	Quantitative	198	16.3 (not specified)	60% females	Education
Halcón, 2003	Antigua, Barbados, Bahamas, BVI, Dominica, Grenada, Guyana, St. Lucia	Multiple	Author developed/adapted questionnaire	Quantitative	15695	10 to 18	61% females	Education

Bhugra, 2003	Trinidad and Tobago, Barbados	Disordered Eating Behaviours and Attitudes	Bulimia Investigatory Test, Edinburgh (BITE); DSM-III-R Bulimia Diagnostic Interview	Quantitative	362	13 to 19	100% females	Education
Pottinger, 2003	Jamaica	Suicidality	Case notes (DSM criteria)	Quantitative	57	12 (2.61)	60% females	Healthcare
Naidu, 2002	Trinidad & Tobago	Stress	Dental Environment Stress (DES)	Quantitative	94	22 (not specified)	55% females	Education
Baboolal, 2002	Trinidad and Tobago	Multiple	Case notes (DSM IV criteria)	Quantitative	32	24.9 (2.83)	50% females	Education
White, 2002	Jamaica	Disordered Eating Behaviours and Attitudes	Author developed/adapted questionnaire	Quantitative	24	18.86 (not specified)	90.91% females	Healthcare
Lambert, 2001	Jamaica	Multiple: behavioural and emotional problems	Child Behaviour Checklist; Jamaican Teacher's Report Form	Mixed Methods	78	8.7 (1.59)	Unclear	Education
Durbrow, 2000	St. Vincent and the Grenadines	Multiple: anxiety and attention problems	Middle Childhood-Home Inventory; Learning Behaviours Scale; Revised Behaviour problem Checklist	Quantitative	61	5 to 14	69% males	Education
Foster-Williams, 2000	Jamaica	Psychological distress	General Health Questionnaire (GHQ); Chronicity and General Health Questionnaire; Social Adjustment Scale - Modified	Quantitative	20	16.5 to 18	65% females	Healthcare
Lambert, 1999	Jamaica	Behaviour and conduct problems	Jamaican Youth Checklist (JYC)	Quantitative	864	6 to 18	Unclear	Education
Perks, 1999	St. Lucia	Depressive symptoms	Reynolds Adolescent Depression Scale (RADS); Reynolds Child Depression Scales (RCDS);	Quantitative	60	9 to 17	60% females	Education
Hilton, 1997	Jamaica	Multiple	General health Questionnaire (GHQ); DSM; Modified social adjustment scale; Psychiatric assessment schedule	Quantitative	89	18 to 20	50.56% females	Healthcare
Deosaran, 1997	Trinidad and Tobago	Behaviour and conduct problems	Author developed/adapted questionnaire	Quantitative	486	<18	75.31% males	Community Group Home
Foster-Williams, 1996	Jamaica	Stress	Questionnaire developed by the University of Texas.	Quantitative	180	University students	50.56% females	Education
Lambert, 1994	Jamaica	Behaviour and conduct problems	Jamaican Teacher Check List (JTCL)	Quantitative	359	6 to 11	50.7% females	Education
Richardson, 1989	Barbados	Personality	Personality Priorities Inventory for Adolescents	Quantitative	194	15.6 (0.29)	58.76% females	Education
Lambert, 1989	Jamaica	Multiple	Clinician developed questionnaire (DSM- criteria)	Quantitative	320	6 to 17	50% females	Healthcare
Payne, 1988	Barbados	Anxiety/Fear	Author developed/adapted questionnaire	Quantitative	657	12 to 15	53.27% females	Education
Burke, 1980	Jamaica	Behaviour and conduct problems	Not applicable	Quantitative	28	Mode 16 or 17	100% males	Healthcare

Wray, 1976	Jamaica	Multiple	Clinical interview (DSM criteria)	Quantitative	154	10 (3)	57.7% males	Healthcare

Note: Table is sorted in descending order according to the year of publication.
